# Focused-Attention Meditation Improves Flow, Communication Skills, and Safety Attitudes of Surgeons

**DOI:** 10.3390/ijerph19095292

**Published:** 2022-04-27

**Authors:** Hao Chen, Chao Liu, Fang Zhou, Xin-Yi Cao, Kan Wu, Yi-Lang Chen, Chia-Yih Liu, Ding-Hau Huang, Wen-Ko Chiou

**Affiliations:** 1 School of Film and Communication, Xiamen University of Technology, Xiamen 361021, China; haochen19606@163.com; 2 Business Analytics Research Center, Chang Gung University, Taoyuan 33302, Taiwan; victory666666@126.com (C.L.); kan@gap.cgu.edu.tw (K.W.); 3 School of Journalism and Communication, Hua Qiao University, Xiamen 361021, China; 4 Department of Economic and Management, Suzhou Vocational Institute of Industrial Technology, Suzhou 215000, China; zhouf@siit.edu.cn; 5 Clinical Neurocognitive Research Center, Shanghai Key Laboratory of Psychotic Disorders, Shanghai Mental Health Center, Shanghai Jiao Tong University School of Medicine, Shanghai 200030, China; rekixinyicao@163.com; 6 Department of Orthopaedic Surgery, Chang Gung Memorial Hospital, Taoyuan 33302, Taiwan; 7 Department of Industrial Engineering and Management, Ming Chi University of Technology, New Taipei 24301, Taiwan; ylchen@mail.mcut.edu.tw; 8 Department of Psychiatry, Chang Gung Memorial Hospital, Taipei 10507, Taiwan; liucy752@cgmh.org.tw; 9 Institute of Creative Design and Management, National Taipei University of Business, Taoyuan 22058, Taiwan; hau1012@gmail.com; 10 Department of Industrial Design, Chang Gung University, Taoyuan 33302, Taiwan

**Keywords:** focused-attention meditation, flow, communication skills, safety attitude, clinical adverse events, patient safety

## Abstract

Objective: Patient safety is a worldwide problem and a focus of academic research. Human factors and ergonomics (HFE) is an approach to improving healthcare work systems and processes. From the perspective of the cognitive ergonomics of HFE, the aim of this study is to improve the flow level, communication skills, and safety attitudes of surgeons through focused-attention meditation (FAM) training, thus helping to reduce adverse clinical events. Methods: In total, 140 surgeons were recruited from three hospitals in China and randomly divided into two groups (FAM group and control group). The FAM group received 8 weeks of FAM training, while the control group was on the waiting list and did not receive any interventions. Three scales (WOLF, LCSAS, and SAQ-C) were used to measure the data of three variables (flow, communication skills, and safety attitude), respectively, at two times, before and after the intervention (pre-test and post-test). The incidence of adverse events during the intervention was also collected for both groups. Results: The ANOVA results showed that all three variables had a significant main effect of time and significant interactions between time and group. The independent-sample T-test results showed that the incidence of adverse events during the intervention was significantly lower in the FAM group than in the control group. Conclusions: The intervention of FAM could significantly improve surgeons’ flow levels, communication skills, and safety attitudes, potentially helping to reduce adverse clinical events.

## 1. Introduction

Patient safety and medical errors have become a worldwide issue and a focus of academic research. The focus and scope of research is broad, ranging from analyzing the causes of medical errors and shortcomings in the clinical process to testing strategies or measures for improvements in patient care and management [1]. Over the past decade, significant effort and resources have been devoted to preventing medical errors and improving patient safety. Human factors and ergonomics (HFE) are key systems for improving the quality of patient care and patient safety [2]. Past studies have shown that many patient safety incidents are related to a lack of attention to human factors and ergonomics (HFE) in the design and implementation of technology, processes, workflows, jobs, teams, and socio-technical systems [3]. Experts and scholars have also designed and developed a series of products, standards, and systems for HFE in healthcare [4].

However, there are still many phenomena that endanger patient safety; medical accidents also occur occasionally, and increasing evidence shows that human factors are the key features of adverse events [4]. In complex healthcare systems, it is inevitable that even experienced, motivated, skilled, and reliable individuals make mistakes because, due to the human factor, mistakes usually occur when systems and technologies do not match the characteristics of the person [5]. Therefore, from the perspective of the cognitive ergonomics of HFE, this study conducted psychological interventions to find a simple and easy method to train surgeons to better match their own characteristics and skills with external challenges, improve their concentration and engagement at work, and enhance their communication skills and safety attitudes.

### 1.1. Focused-Attention Meditation

Meditation consists of a set of mental exercises used to develop a cognitively and emotionally balanced mind, and its development and practice date back 4000 years [6]. Over the past 50 years, there has been increasing interest in meditation, largely because of its effectiveness in improving emotional regulation [7]. Meditation is often conceptualized as a series of attentional and emotional regulation exercises [8]. There are many ways to practice meditation, and a common way to classify this large family of practices is based on what meditators do from their first-person perspective: focused-attention meditation (FAM) and open-monitoring meditation (OMM) [9]. While FAM has a clear focus on objects such as breathing, OMM practice (e.g., mindfulness meditation) has no clear focus, and the task is to be constantly aware of what is happening and to return to this monitoring state when drawn to something else [10]. The purpose of FAM is to resist the outside world and no longer receive external information, while that of OMM is to be inside, to treat and restore the current self-state [9]. FAM emphasizes attention maintenance and aims to consciously induce a state of relaxation, while OMM focuses on monitoring attention and emphasizes acceptance without judgment [9,11]. In this study, FAM was chosen as the intervention method because FAM simply focuses on the improvement of attention, while OMM maintains the awareness and monitoring of internal and external stimuli while maintaining attention.

During FAM, the meditator brings her/his attention to an object, such as breathing, and then uses this attention to monitor whether the attention is still there; once the meditator realizes that attention has strayed, he or she returns to the object in focus, minimizing any further mental elaboration [12]. It is worth noting that this is in contrast to our habitual reactions, in which we tend to feel frustrated by our inability to stay focused, leading to feelings of disgust [13]. By aversive, we refer to emotional states experienced as aversive, while pleasant refers to the opposite. In other words, meditation can reduce aversive feelings by developing mental habits that reduce this amplification and catastrophizing process, and this in itself may be one of the most important beneficial mechanisms through which FAM relieves mood and stress [14,15].

Moye and van Vugt [16] found that the ability to maintain attention was significantly improved after FAM practice than before, and speculated that the improvement in mood regulation observed after meditation was due to the ability to maintain focus. A series of studies by Chan et al. has confirmed that FAM affects a series of attention-related learning and cognitive processes. FAM establishes a state of enhanced cognitive control, and enhances the effect of top-down control on sequence-learning based on the control characteristics of attention [12]. FAM may be associated with enhanced cognitive control to facilitate the development of a more efficient stimulus–response process compared to other forms of attentional task induction [8,10]. Many studies have explained the mechanism through which FAM enhances attention from a neurological perspective. Irrmischer et al. [17] found that the effect of FAM on attention was associated with greater control, and FAM strongly suppressed the long-range temporal correlations (LRTC) of neuronal oscillations relative to eyes-closed rest. The ability to reduce LRTC during meditation increased, which was associated with maintaining focus [17]. Manna et al. [18] found that the functional reorganization of brain activity patterns for attention and cognitive monitoring occurred during FAM practice. In a study by Yoshida et al. [14], the FAM group showed significantly higher P3 amplitude and shorter response time to target T stimulus during the task; by contrast, no such correlations were observed in the control group. These findings provide direct evidence of the effectiveness of FAM training.

Surgeons need to maintain a high level of concentration while performing surgery, and they also need channels to relieve the intense stress and negative emotions involved in such a high-intensity job; therefore, FAM practice may help them.

### 1.2. Flow and Focused-Attention Meditation

Flow refers to a mental state experienced by being fully engaged and deeply immersed in the task or activity at hand, in which people are fully engaged in the activity and gain many positive experiences [19]. Csikszentmihalyi [20] believed that flow is a positive emotion and experience related to a task. Flow refers to the mental state in which an individual uses his or her skills to complete a series of challenges and achieve a goal. In this process, individuals constantly receive positive feedback and adjust their behavior according to this feedback [21]. Flow can prompt an individual to show a strong interest in an activity or thing and motivate them to participate in it [22]. What FAM and flow have in common is a high degree of concentration. FAM is an effective training method for concentration. Individuals with higher levels of concentration are more likely to enter the flow state [23].

Bakker [24] applies flow to work situations. According to flow’s attribute description, flow is most likely to occur when the challenge of a situation is balanced with a person’s ability to cope with this challenge [20]. Analogously, in work situations, employees experience work-related flow when their work needs are matched with their skills [25]. Work-related flow is a peak experience generated by individuals at work, characterized by clear goals, focus, and matching of skills with challenges [24]. The nature of surgeons’ work makes it easier for them to experience flow. First, the goal of the surgeon’s job is clear: to treat patients and remove and repair diseased tissue. Secondly, surgeons need to maintain a high degree of concentration in their work. Thirdly, the complexity of surgery poses significant challenges, requiring surgeons to constantly improve their skills.

### 1.3. Communication Skills and Focused Attention

Communication and teamwork can be complex skills to apply in the operating room, as the members of operating-room teams vary by type of surgery [26]. Communication is the process of information exchange between people. Surgery is an important means of eradicating or effectively treating some diseases, and it is the embodiment of medical technology and medical skill [27]. Effective communication between surgeons and other medical workers and between surgeons and patients is the basis for improving medical quality and achieving the expected results of surgery [28,29]. Therefore, surgeons need to have good communication skills. Surgery requires the surgeon to work with nurses and anesthesiologists. Different medical specialties have different working styles, and surgeons must have a high degree of responsibility, respect, and understanding of the nature and characteristics of the work of other medical staff [30]. Surgeons should establish a harmonious working environment, in which all colleagues display a positive working attitude, support and cooperate with each other, do not shirk responsibility, and analyze and solve problems directly, so as to ensure smooth operation and better embody the idea of patient-centered medicine [31].

When people communicate, they mobilize an ability to coordinate their attention with that of others, which is called joint attention [32]. Joint attention is usually based on visual attention to define social coordination following another person’s gaze, adopting common reference points to operate, and on using the direction of one’s own gaze or position to determine, with another person, the potential of common reference points [33]. According to the theory of joint attention, as visual attention ability improves, visual attention develops into the ability to coordinate mental attention with others [32]. Increased focus helps to promote joint attention, which, in turn, helps to improve communication [34]. Many previous studies have also confirmed the effect of attention on communication skills. Karnieli-Miller et al. [35] found that the higher the concentration levels of medical students, the better their clinical communication skills. The reason is that in communication, we must always pay attention to the facial expression, voice, intonation, and emotional changes of the communication object, and focus on practice involves awareness of the moment [36]. People with high levels of focus are more effective at capturing details and have keen insight into emotions and affect, so highly focused people are more effective at clinical communication [37].

### 1.4. Patient Safety and Safety Attitude

The World Health Organization (WHO)’s World Alliance for Patient Safety and the International Patient Safety Goals (IPSG) recognize patient safety as part of a global strategy aimed at minimizing adverse events and eliminating preventable harm in healthcare systems [38]. Patient safety is defined as “the avoidance and prevention of patient injury or adverse events resulting from medical services” [39]. Patient safety should be guaranteed in everyday practice because it improves the quality of care, ensures correct diagnosis, prevents nosocomial infections and medication errors, and ultimately provides correct management [40,41]. The growing recognition that patient safety is a key to the quality of healthcare products has contributed to the importance of a patient safety culture in healthcare organizations [42]. An organization’s safety culture is the product of individual and group values, including attitudes, perceptions, competencies, and behavior patterns that determine commitment to the organization’s health and safety management [43]. Safety attitudes include six major patient safety factors, namely, teamwork atmosphere, safety atmosphere, job satisfaction, management perception, working conditions, and stress perception [44]. Safety attitudes help to identify weaknesses that may exist in clinical settings and facilitate quality improvement interventions and reductions in medical errors [45].

In summary, communication, work stress, and job satisfaction are important factors influencing safety attitudes [44]. FAM can help relieve stress, regulate mood, and reduce work stress [14,15]. Improved concentration makes surgeons more focused on the work at hand, less likely to be distracted, more likely to enter the state of flow, experience pleasure, and, thus, improve job satisfaction. Increased focus also improves communication skills, which in turn helps achieve effective teamwork [37]. Therefore, FAM practice may be a simple and effective intervention to help improve the safety attitudes of surgeons.

### 1.5. Research Purpose and Hypotheses

The purpose of this study is to improve flow levels, communication skills, and safety attitudes among surgeons through FAM intervention.

Based on the above literature, this study proposes the following hypotheses:

**Hypothesis** **1** **(H1).** *FAM practice will significantly improve the flow level, communication skills, and safety attitudes of subjects in the experimental group.*

**Hypothesis** **2** **(H2).** *Through the intervention of FAM practice, the experimental group will show better patient safety performance than the control group*.

## 2. Method

### 2.1. Participants

Subjects in this study were recruited from the surgical departments of three hospitals in China, and a total of 140 qualified subjects participated in and completed the study. The subjects were randomly divided into two equal groups, the FAM group and the control group, with 70 participants in each group. Table 1 shows the demographic information of the subjects. To avoid the effect of gender on the results, we matched the gender composition between the two groups, and there was no significant difference between the two groups in age composition, sex ratio, or other demographic factors.

### 2.2. Instruments

Work-related Flow Inventory (WOLF). This is a 12-item self-report scale developed by Bakker [24]. Based on the conceptual characteristics of work-related flow, this scale consists of three dimensions (each dimension contains four items): concentration on task, clear goals, and challenge–skill balance. A five-point Likert scale was used to measure the levels of flow characteristics experienced, ranging from 1 (never) to 5 (always), with higher scores indicating higher work-related flow levels. The Chinese version of WOLF used in this study was translated by Gu et al. [46]. The Cronbach’s alpha of WOLF in this study was 0.84.

Liverpool Communication Skills Assessment Scale (LCSAS). This scale was developed by Humphris and Kaney [47] to measure the communication skills of doctors. The scale has 12 items in total, including five dimensions: introduction, nonverbal behavior, respect and empathy, questioning, and giving information. A four-point scale was used: 1 = unacceptable, 2 = poor, 3 = acceptable, and 4 = good. The scale is widely used in clinical practice to assess physicians’ communication skills, and the literature supports LCSAS as a reliable tool with acceptable reliability and validity [48]. The Chinese version of LCSAS used in this study was translated by Liu et al. [49]. The Cronbach’s alpha of LCSAS in this study was 0.89.

Safety Attitudes Questionnaire-C (SAQ-C). The scale was developed by the University of Texas and has a reliability of 0.9 [50]. SAQ has 32 items across five dimensions: teamwork climate, safety climate, job satisfaction, perception of management, and working conditions. The questions were answered on a five-point Likert scale (1 = strongly disagree, 2 = slightly disagree, 3 = neutral, 4 = slightly agree, and 5 = strongly agree). SAQ-C is a reliable tool for eliciting provider attitudes about medical safety [51]. The Chinese version of SAQ-C used in this study was translated by Lee et al. [52]. The Cronbach’s alpha of SAQ-C in this study was 0.91.

Clinical Adverse Events (CAE). We examined the following five kinds of higher-frequency adverse event in clinical operation: surgical site infection, urinary tract infection, ventilator-associated pneumonia, medication errors, and dressing mistakes [53]. This was a self-report scale; the participating surgeons rated the frequency of all adverse events that occurred during the trial. The frequency of adverse events was described using a six-point Likert scale from 1 (daily) to 6 (never), with a higher score indicating a lower incidence of adverse events.

### 2.3. FMA Practice

Subjects in the FAM group underwent 55 min of FAM practice intervention three times a week for a total duration of 8 weeks. The practice site was located in a meditation yoga practice room, which was spacious, quiet, and easily accessible. Due to the busy work schedule of surgeons, the FAM meditation practice was conducted every night on weekdays and all day on weekends. Subjects chose to participate three times a week according to their own schedule. The meditation practice was conducted by two instructors with more than 3 years of experience in FAM instruction each. The instructors did not know the purpose of the study, the specific arrangement of the experiment, and which participants were involved in the study in each activity. Each meditation practice was observed and recorded by a researcher who did not interfere with the practice.

Each 55-min meditation practice included the following procedures: A 10-min period of instructor guidance, 35 min FAM practice (with a 5-min break), and 10 min exchanging experiences and discussion. The instructor taught meditation techniques in the 10 min guidance session prior to meditation, and helped participants relax and ease into the meditation state. The specific operation methods of FAM for 30 min are as follows: (1) Adjust to a comfortable sitting position to relax the body and mood; (2) choose a point of focus, which can be breathing or chanting mantras, or anything else; (3) focus all your attention on the point, feel and observe it, and let go of other thoughts and feelings; (4) if there is a distraction, when you notice it, transfer your attention to the point and continue to observe and feel it. After completing the 15-min meditation, take a 5-min break and meditate for the next 15 min. The 10-min discussion time after the meditation was used for participants to communicate about the meditation, so that the instructor could understand the participants’ experience of meditation and answer their questions accordingly.

### 2.4. Procedue

A 2 (group) × 2 (time) parallel randomized controlled trial design was used in this study, in which the intervention condition was FAM practice and the control condition was waiting. The first test time node was the baseline before the experiment, and the second test time node was the level after the intervention.

We advertised for FAM practice on the internal network of three hospitals in China. The recruiting advertisement said that there was a study on meditation to improve concentration and offered a free meditation-training program to help improve concentration, regulate emotions, and reduce work stress. Interested surgeons were invited to sign up.

**Figure 1:**
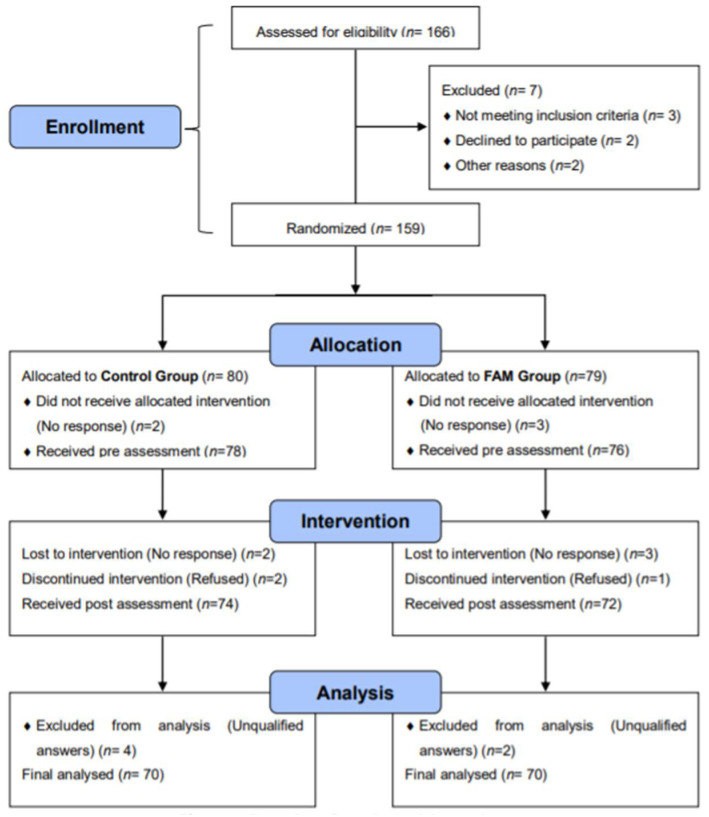
Procedure flow chart of the study.

FAM training was free for 8 weeks and aimed to improve concentration, mood and relaxation. The sample size was determined by G*Power. Two groups of four scales were used for the measurements, at α = 0.05 and 80% power, f = 0.3, which was the medium effect size of ANOVA with repeated measures. The recommended sample size was 100, and we expected an attrition rate of 20% from pre-test to post-test, so we attempted to recruit at least 120 participants. After screening, our pre-test sample included 154 participants, of whom 140 completed the post-test. Figure 1 depicts the experimental process and the flow chart of the study.

The inclusion criteria of subjects were: (1) Doctors in surgical departments; (2) at least 2 years of related working experience; (3) aged between 25 and 55; (4) agreed to participate in this study and signed informed consent. Exclusion criteria were: (1) History of mental illness or mental disorder; (2) have taken psychotropic drugs in the past 2 years, or are currently using psychotropic drugs; (3) have received any form of psychological intervention in the past 2 years; (4) have any form of meditation training experience.

In this study, random sequence codes generated by SPASS 22 software were used for random grouping. A list of codes labeled “Group A” and “Group B” was given to participants who were randomly assigned to either group A or group B in a 1:1 allocation ratio. Group A was the experimental group and immediately started FAM practice, while group B was the control group waiting to participate in the next practice. Subjects, data analysis staff, and other researchers participating in the study were not informed of the details of the grouping and the corresponding relationships before the completion of the experimental procedure and data analysis. The study was therefore double-blind.

Each participant signed a subject consent form to ensure they understood and agreed to participate in the study before proceeding. Participants were informed that they had the right to terminate their participation in the study without any reason, at any time, without prejudice against their legal rights and interests. Researchers could also pause the study if necessary. The purpose of the pre-test was to establish a baseline for the experiment. Prior to the start of the entire intervention program, the subjects signed the subject consent and conducted the scale evaluation of the pre-test. The pre-test included demographic information and three scales, WOLF, LCSAS, and SAQ-C, which took approximately 40 min to answer. The total duration of the intervention was 8 weeks. There was a post-test at the end of the entire intervention, at which point the participants completed three scales (WOLF, LCSAS, and SAQ-C) again. Furthermore, since participants were asked to recall the approximate frequency of adverse events during the 8-week intervention period based on their memory, CAE data were collected. To encourage participants to truthfully report their rate of adverse events, they were allowed to use nicknames instead of their real names. The time to answer the post-test questionnaire was also approximately 40 min. Finally, the researchers explained to the participants the real purpose of the study and thanked them. This study was approved by the Ethics Committee of Chang Gung University and in accordance with the ethical guidelines.

### 2.5. Data Analysis

In this study, IBM SPSS 22 software was used for statistical analysis. Descriptive statistics were used to describe the characteristics and distribution of subjects’ demographic data and experimental data. ANOVA with repeated measures was used to compare the differences before and after intervention and the differences between groups. Independent-sample T test was used to compare the differences in the rate of adverse events between the two groups. The significance level was set at 0.05.

## 3. Result

**Figure 2:**
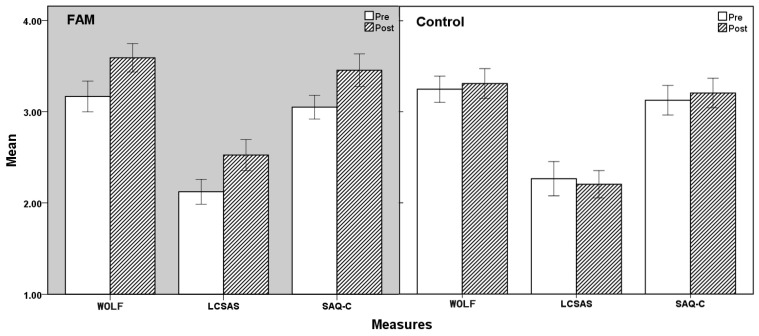
Comparison between FAM group and control group. Errors bars: 95% confidence interval; CAE: clinical adverse events; FAM: focused-attention meditation; LCSAS: Liverpool Communication Skills Assessment Scale; SAQ-C: Safety Attitudes Questionnaire-C; WOLF: work-related flow inventory.

This study consisted of three 2 (group type: FAM, control) × 2 (time: pre-test, post-test) ANOVAs with repeated measures. The results of descriptive statistics and paired-sample T tests are shown in Table 2, and the ANOVA results are shown in Table 3 and Figure 2. The *p*-values of the Box’s test, Mauchly’s test, and Levene’s test were all greater than 0.05, indicating that these data were suitable for ANOVA.

In terms of flow, communication skills, and safety attitudes, there were a significant main effect of time and significant interactions between time and group, but no significant main effect of group (see Table 3). These results indicate that the FAM intervention significantly improved participants’ flow levels, communication skills, and safety attitudes, so Hypothesis 1 was supported.

The incidence of CAE during FAM intervention was reported post-test (see Table 1). The independent-sample T-test results showed a significant difference in CAE scores between the two groups (*p* = 0.003, Std. error = 0.067). The CAE score of the FAM group was significantly higher than that of the control group, indicating that the incidence of adverse events within 8 weeks of the experimental intervention was lower in the FAM group than in the control group, thus supporting Hypothesis 2.

## 4. Discussion

### 4.1. FAM Improves Flow Levels

The results of this study found that the intervention of FAM significantly improved the flow level of the subjects. FAM and flow experience have some common characteristics, in that they both emphasize the importance of being present [54]. Flow requires a very concentrated moment of attention and unintentionally focuses on a specific task [55]. FAM may create the basic conditions for flow experience. For this reason, many scholars suggest that focusing on the present is an effective strategy for achieving flow. Csikszentmihalyi [20] explained that flow experience is due to exceptionally strong concentration within a limited stimulus area, and present consciousness is the common feature of focused meditation and flow experience. Turnbull et al. [56] found that subjects with higher concentration levels were more likely to experience flow. Their study suggests that concentration may be a catalyst for flow, that the changes in attention experienced by participants are positively correlated with changes in flow, and that FAM interventions can effectively promote flow experiences. Some evidence of causality in the attention–flow relationship was obtained in studies of athletes, which found that FAM interventions may increase flow experiences [57]. Athletes in the FAM training program experienced higher levels of flow than before, as well as higher flow levels than athletes who did not take part in meditation training [58].

Some scholars explain this phenomenon from the perspective of neurocognitive function. Weber et al. [59] argued that flow experience is related to the synchronization of the attention network and the reward network. In flow experience, the attention network associated with flow experience discharges synchronously with the reward network. Klasen et al. [60] also found, in a fMRI (functional magnetic resonance imaging) study of flow experience, that as flow experience decreased in video game players, the synchronization between the attention network and the reward network also weakened, further confirming that flow experiences are related to the synchronization between the attention network and the reward network.

### 4.2. FAM Improves Communication Skills

The results of this study suggested that the FAM intervention significantly improved the subjects’ communication skills. First, attention can indirectly influence clinical communication skills through cognitive reappraisal. Attention is the awareness of the body’s feelings, emotions, mental representations, perceptual experiences, and cognitive reappraisal [37]. This awareness enhances concentration and cognitive functions, reduces automated responses, relieves stress, and reduces redundant and negative thinking modes, thus reducing the difficulty of emotional adjustment and increasing the use of cognitive reappraisal strategies [37]. Secondly, according to the theory of social emotional choice, when individuals are aware of the time limitations, their emotional goals take precedence, that is, they focus their attention on the present and have positive cognitive preferences, so as to produce positive cognitive processing [61]. Emotional self-regulation and control is the foundation of communication with patients, and surgeons may encounter in the process of clinical operation all kinds of problems and frustrations, such as puncture failure or medication errors and other adverse clinical events [62]. When doctors with high concentration skills show negative emotions, they can flexibly use emotion regulation strategies to adjust their psychological state through cognitive reappraisal, so as to fundamentally improve their ability to communicate with patients [63,64].

### 4.3. FAM Improves Safety Attitudes

This study found that FAM intervention significantly improved the safety attitudes of surgeons. At present, society places increasing demands on the professional and personal ability of surgeons [65]. Surgeons have to face increasing pressure and challenges. If they are in this pressured working state for a long time, they are prone to negative emotions, which make them unable to devote themselves to their work in the best state and increase the risk of medical safety issues [66]. Studies have shown that FAM can help surgeons focus on the needs of patients, improve practical problem-solving and awareness of their own state, help reduce stress, and reduce adverse medical events [67].

First, training helps improve surgeons’ concentration and emotional regulation, enabling them to focus on the work at hand, effectively deal with various disturbances and efficiently complete tasks. When surgeons face patient complaints, accusations can be dealt with in a peaceful state of mind and with an optimistic attitude to solve the problem [68]. Second, awareness training can reduce the pressure surgeons place on themselves, which can enable surgeons to discover their inner sense of time-pressure, release their dissatisfaction, maintain a peaceful mindset, and improve their sense of professional interest and reduce job burnout at work, so as to help them to develop a more positive, healthy attitude to work and improve their perceptions of and attitudes towards stressful events [69]. In addition, training improves subjects’ communication skills, which, in turn, enhances teamwork among healthcare professionals and facilitates the formation of supportive relationships among team members. Through mutual assistance, mutual support and reminders among team members, professionals identify problems at work in a timely manner and deal with them with the strength of their team [70]. Effective communication and assistance between team members can effectively improve work efficiency and safety, create a positive safety culture, and improve safety attitudes [71].

### 4.4. FAM May Help Reduce Adverse Events

Errors in surgeons’ work involve perceptual errors, judgment errors, and action errors, which are the direct causes of adverse clinical events [72]. Perceptual errors are usually caused by inadequate psychological preparation, excessive emotional tension and paralysis, low perceptual level, distraction, and slow reactions, among others. Perception errors, lack of experience, and poor resilience often lead to errors in judgment [73]. Perception errors and judgment errors further lead to operational errors, resulting in adverse events [74]. During a long operation, as the workload increases, the surgeon’s perception process undergoes a series of changes.

Attention is a mental state that always accompanies the cognitive process [75]. It seems to be a kind of selective filter for concentration, which enables people to input information selectively and focus their attention on the information to be input, processed, extracted, and output [75]. However, people’s ability to process information at the same time is limited. If the input information is too large, people’s thinking enters a state of chaos, and, if coupled with low quality of information or interference by objective conditions, concentrated attention is reduced [75]. In specific stages of surgery, due to surges of information, attention capacity is limited, so that surgeons experience difficulties in their distribution and transfer of attention [76]. The narrow scope of attention and the interference of irrelevant stimuli place information beyond the attentional capacity of surgeons [53]. Obviously, in such a state, surgeons’ cognitive processes are disrupted.

Working under high load can make surgeons nervous and anxious, which makes it difficult for them to carry out operations as normal [12]. When emergent, unexpected, complex, urgent, and dangerous emergency situations appear, they not only increase the workload of surgeons, but also increase their psychological load [77]. At this point, the surgeon’s mind is often too tense, and their mood can become extremely unstable [78]. When an emergency exceeds the surgeon’s ability to respond, it can lead to a sharp decline in their ability to work, often in the form of decreased perception, reduced attention span, undesired omissions, and even the inability to determine what to do and to “turn a blind eye” [79].

Mind-wandering is the act of diverting attention from work tasks to unrelated things. Spontaneous mind-wandering is often associated with self-reflective states that lead to negative processing of the past, worries and fantasies about the future, and the disruption of primary task performance [80]. While the key of FAM is concentration, FAM can help surgeons to learn to monitor when mind-wandering occurs, which can improve the performance of tasks that require sustained attention and intense focus [10]. FAM can reduce mind-wandering, improve cognitive performance, improve participants’ ability to maintain attention in the presence of external distractions, help improve concentration on tasks, and reduce performance degradation due to task-unrelated distractions [81]. Meditation training increases awareness and decreases mind-wandering, which results in optimal actions and fewer adverse events only when the subject is fully engaged in the present moment [82].

### 4.5. Research Limitations and Future Studies

This study explores the positive effects of interventions on improving flow, communication skills, and safety attitudes among surgeons, and draws some innovative and practical conclusions. However, due to reasons of manpower, material resources, and time, there are also the following limitations: (1) The subjects of this study were all surgeons. Whether the results of this study can be extended to other medical and health practitioners needs further investigation and verification; and (2) in this study, self-reported questionnaires were used, and the frequency of reported adverse events may be reduced when surgeons perform self-evaluation.

Based on the limitations of this study, the following suggestions are put forward for future research: (1) Future studies should improve and enrich data collection and measurement tools and integrate multiple evaluation methods, such as third-party evaluation and behavioral observation. Furthermore, future research should ensure that the data reflect the situations of subjects more truly and effectively, and include the performance of a more comprehensive evaluation of various variables to better meet the research needs; and (2) the structural equation model and other investigation and research methods should be used to further explore the relationships between variables and clarify the deep influence mechanisms acting on each variable.

## 5. Conclusions

This study found the following. (1) FAM intervention can significantly improve flow, communication skills, and safety attitudes among surgeons. (2) FAM intervention may help to reduce the rate of adverse clinical events. The specific mechanism observed in this study was as follows: (1) FAM enhanced the ability of surgeons to control their own attention; and (2) FAM can help relieve negative emotions, such as stress and anxiety. (3) FAM improved the flow level of the subjects and made them more likely to experience flow at work. The pleasant experiences generated by flow further offset the negative emotions of the subjects, and stimulated work motivation. (4) FAM improves subjects’ communication skills and safety attitudes, improves teamwork, and contributes to a safe working atmosphere. The above points may help surgeons reduce errors in perception, judgment, and action in their work, thus reducing the incidence of adverse clinical events.

## Figures and Tables

**Table 1:** Demographic characteristics of participants.

Characteristic	Total	FAM Group	Control Group
Age (SD)	40.56 (7.26)	39.19 (7.78)	41.94 (7.62)
Male (%)	103 (73.6%)	50 (71.4%)	53 (75.7%)
Female (%)	37 (26.4%)	20 (28.6%)	17 (24.3%)

Note. No demographic characteristic was significantly different among the two groups. FAM: Focused-attention meditation.

**Table 2:** Results of descriptive statistics.

Group	Measure	Mean (SD)	
		Pre	Post
FAM	WOLF	3.166 (0.709)	3.590 (0.652)
	LCSAS	2.121 (0.670)	2.523 (0.713)
	SAQ-C	3.049 (0.648)	3.454 (0.751)
	CAE		5.155 (0.383)
Control	WOLF	3.247 (0.602)	3.310 (0.688)
	LCSAS	2.264 (0.688)	2.203 (0.631)
	SAQ-C	3.127 (0.681)	3.205 (0.682)
	CAE		4.957 (0.402)

Note. CAE: clinical adverse events; FAM: focused-attention meditation; LCSAS: Liverpool Communication Skills Assessment Scale; SAQ-C: Safety Attitudes Questionnaire-C; WOLF: work-related flow inventory.

**Table 3:** Results of ANOVA.

Measure	Variable	F	*p*	η^2^
WOLF	Time **	9.691	0.002	0.066
	Group	1.539	0.217	0.011
	Time × Group *	5.332	0.022	0.037
LCSAS	Time *	4.249	0.041	0.030
	Group	1.233	0.269	0.009
	Time × Group **	7.833	0.006	0.054
SAQ-C	Time **	10.013	0.002	0.068
	Group	1.053	0.307	0.008
	Time × Group *	4.545	0.035	0.032

Note. * *p* < 0.05; ** *p* < 0.01. LCSAS: Liverpool Communication Skills Assessment Scale; SAQ-C: Safety Attitudes Questionnaire-C; WOLF: work-related flow inventory.

## Data Availability

The data in the research are not publicly available. If there is a reasonable request for data viewing and use, the corresponding author can be contacted.
